# Synthesis of Highly Polymerized Water-soluble Cellulose Acetate by the Side Reaction in Carboxylate Ionic Liquid 1-ethyl-3-methylimidazolium Acetate

**DOI:** 10.1038/srep33725

**Published:** 2016-09-20

**Authors:** Jinhui Pang, Xin Liu, Jun Yang, Fachuang Lu, Bo Wang, Feng Xu, Mingguo Ma, Xueming Zhang

**Affiliations:** 1 Beijing Key Laboratory of Lignocellulosic Chemistry, Beijing Forestry University, Beijing 100083, PR China; 2 State Key Laboratory of Pulp and Paper Engineering, South China University of Technology, Guangzhou 510640, PR China

## Abstract

In the present study, we describe a novel one-step method to prepare water-soluble cellulose acetate (WSCA) with higher degree of polymerization values (DP = 650–680) by *in situ* activation of carboxyl group in ionic liquid. First of all, cellulose was dissolved in 1-ethyl-3-methylimidazolium acetate (EmimAc) and reacted with dichloroacetyl chloride (Cl_2_AcCl) in order to make cellulose dichloroacetate. Under various conditions, a series of water soluble products were produced. Elemental analysis and NMR results confirmed that they were cellulose acetate with DS (degree of substitution) values in the range from 0.30 to 0.63. NMR studies demonstrated that Cl_2_AcCl reacted with acetate anion of EmimAc producing a mixed anhydride that acetylated cellulose. Other acylating reagents such as benzoyl chloride, chloroacetyl chloride can also work similarly. 2D NMR characterization suggested that 6-mono-O-acetyl moiety, 3,6-di-O-acetylcellulose and 2,6-di-O-acetyl cellulose were all synthesized and the reactivity of hydroxyl groups in anhydro-glucose units was in the order C-6>C-3>C-2. This work provides an alternative way to make WSCA, meanwhile, also services as a reminder that the activity of EmimAc toward carbohydrate as acylating reagents could be a problem, because the expected acylated products may not be resulted and recycling of this ionic liquid could also be difficult.

Ionic liquids, it is defined as a class of environmentally friendly organic salts with high thermal stability, negligible vapor pressure, wide liquid range, and tunable salvation properties^1^,^2^. Over the past few decades, various applications of ionic liquids have been used as solvents for sustainable organic synthetic processes^2^, as battery electrolytes^3^, for capturing CO_2_^4^, and deconstructing lignocellulosic biomass^5^,^6^. Moreover, it has been known that some ionic liquids exhibited excellent cellulose dissolving ability^7^,^8^, and this initiated a great deal of studies on cellulose dissolution and modifications.

Cellulose is a high molecular weight linear natural polymer with D-glucopyranose units connected through β-(1–4) linkages. It is considered as one of the most promising polymeric resource and exhibits numerous applications as bio-based materials due to its biocompatibility and biodegradability^9^. However, the strong intermolecular hydrogen bonding interactions between units and its crystalline structure make the polymer insoluble in majority of conventional solvents limiting the potential applications of this abundant renewable resources^10^. So far, the dissolution and fabrication of cellulose materials are predominantly performed in NaOH-CS_2_ (viscose process) and *N*-methylmorpholine *N*-oxide (NMMO, Lyocel process). However, these methods have serious drawbacks related to their chemical and thermal stability, environmental impact, and recycling issues^11^. The emerging ionic liquids represents a promising alternative to existing cellulose-dissolving solvents due to their better thermal stabilities relative to NMMO^12^. A quite number of research^11^,^13^,^14^ has been carried out to investigate the potential of these solvents for the homogeneous chemical modifications of cellulose, in which acylation of cellulose using different acylating reagents succeeds with remarkable efficiency.

In order to enhance the solubility and processability of cellulose, modification through substitution reactions of its hydroxyl groups has been extensively carried out^15^. Among these modified cellulose derivatives, cellulose acetate (CA) is one of the most important esters of cellulose, which are obtained by reaction of cellulose with acetic anhydride in the presence of catalyst^16^. Depending on the way it has been processed, cellulose acetate can be used for great varies of applications, such as films, membranes, fibers and cigarettes^17^. Moreover, the solubility of cellulose acetate lies in the degree of substitution (DS). CA with DS of 2–2.5 is soluble in some organic solvents, such as acetone, dioxane and methyl acetate and higher acetylated types are soluble in dichloromethane^18^. In addition, CA may also become water-soluble with the moderately low degree of substitution (0.4–0.9)^19^. At present, it is difficult to prepare such water-soluble cellulose acetate (WSCA) samples directly. In general, the process of preparation of water-soluble CA is fairly complex by a two-step method. Firstly, cellulose with relatively low degree of polymerization value (DP = 200–300) was acetylated, and then the obtained CA was subsequently deacetylated by a complicated hydrolysis process in acidic media^19^. Unfortunately, this deacetylated method seriously damages the structure of cellulose and leads to further degradation of the cellulose. On the other hand, it has been reported that the relative DS values at C-2 and C-3 secondary hydroxyl groups play a key role in the water solubility of cellulose acetate (CA)^20^. However, the distribution of substituents in the anhydroglucose units (AGU) are difficult to control in this hydrolysis process, resulting in lower dissolving ability in water^19^.

In the present work, we attempt to prepare dichloroacetate of cellulose using dichloroacetyl chloride in ionic liquid EmimAc. Surprisingly the obtained product was soluble very well in water because the expected cellulose dichloroacetate should be less polar than cellulose acetate that usually is not water soluble. Elemental analysis and NMR characterization of the product suggested that cellulose acetate was the product instead. More interestingly, the obtained cellulose acetate possessed good water solubility with relatively higher DP values (650–680). For comparison, our study was then extended to include other acylating agents such as chloroacetyl chloride (ClAcCl) and benzoyl chloride. The mechanism for the formation of the unexpected cellulose acetate was demonstrated based on the NMR analysis results.

## Results

The homogeneous modification of cellulose with dichloroacetyl chloride (Cl_2_AcCl) in EmimAc produced, surprisingly, the water-soluble cellulose derivatives. The preliminary analysis by ^1^H NMR suggested that the obtained product might be cellulose acetates with low degree of substitution. In Table 1, the reaction conditions (time and temperatures) and DS values of the modified cellulose derivatives are summarized. First of all, cellulose acetate products with DS values of 0.30 to 0.63 were obtained under various conditions. Increasing the reaction time from 6 to 24 h (70 °C) resulted in an increase of the DS from 0.30 to 0.63. The resultant products became soluble in H_2_O and DMSO when the reaction time reached 6 h at 70 °C. Reactions performed at lower temperature gave rise of products with lower DS (entry 5 and 6). It was noteworthy that these modifications resulted in no degradation as indicated by the DP values (DP = 650–680) of the products that were comparable to that of the control (DP = 655, regeneration from IL without further modification), which was much higher than that from previously published literature^21^. The WACA samples obtained with the relatively high DP are mainly because the solution and modification of cellulose were conducted in IL. Lots of works have been conducted to investigate the dissolution mechanism of cellulose in ionic liquids^22^,^23^,^24^. Zhang *et al*.^24^ revealed that the hydrogen bonding of hydroxyls with the acetate anion and imidazolium cation of EmimAc is the major force for cellulose dissolution, rather than breaking down the degree of polymerisation. ILs are a mild medium for dissolution and reaction medium of cellulose, herein, the obtained CA samples in EmimAC possessed the relatively high DP.

**Figure 1:**
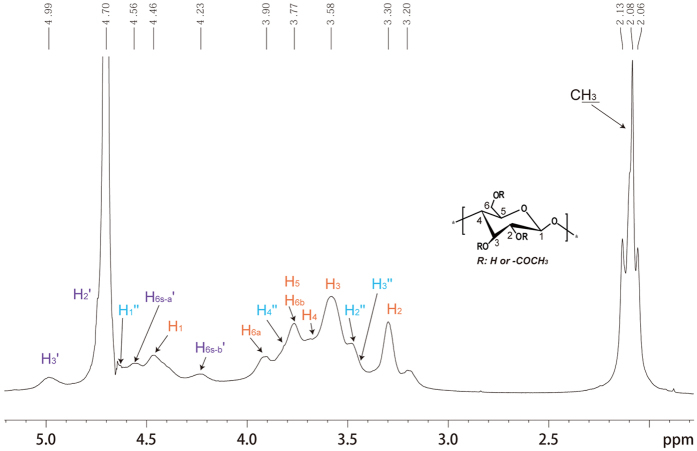
^1^H NMR spectra of sample 4 (DS = 0.63) recorded in D_2_O at 25 °C. Assigned peaks: H colored in rust represents the unsubstituted carbon or partially substituted in glucose units; H colored in purple with mark index′ represents the substituted carbon; H colored in cyan and marked with index′′ represents carbon adjacent to substituted position.

**Figure 2:**
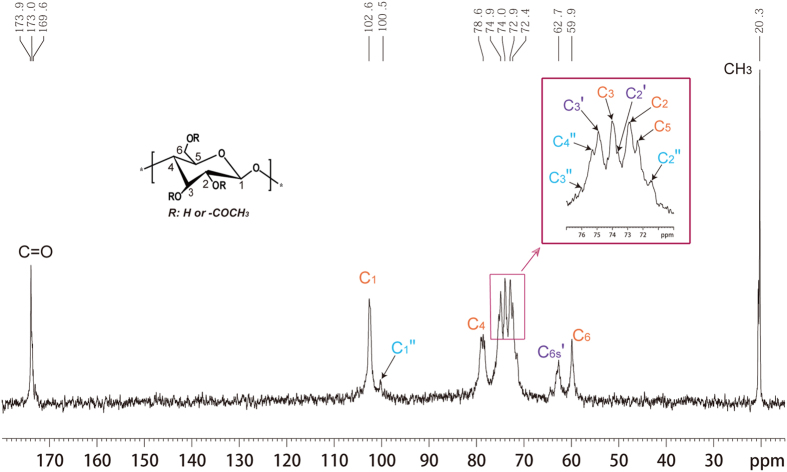
^13^C NMR spectra of sample 4 (DS = 0.63) recorded in D_2_O at 25 °C. Assigned peaks: C colored in rust represents the unsubstituted carbon or partially substituted in glucose units; C colored in purple with mark index′ represents the substituted carbon; C colored in cyan and marked with index′′ represents carbon adjacent to substituted position.

**Figure 3:**
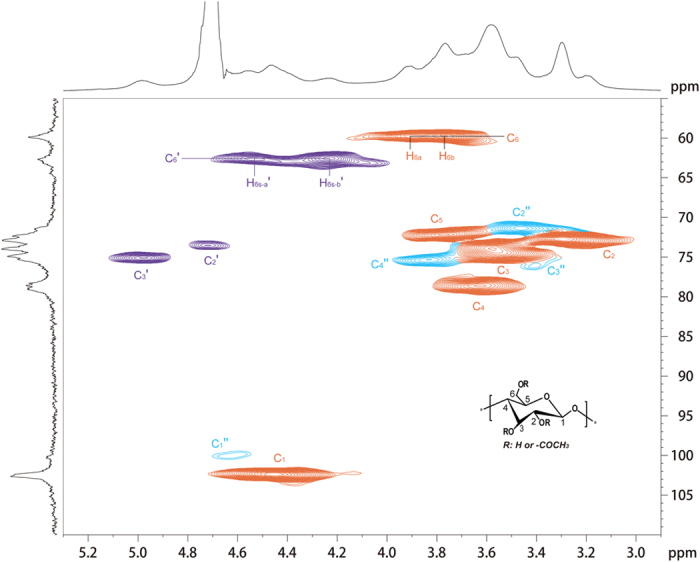
HSQC NMR spectra of sample 4 (DS = 0.63) recorded in D_2_O at 25 °C. Assigned peaks: Contours numbered and colored in rust represents the unsubstituted carbon or partially substituted in glucose units; Contours numbered and colored in purple with mark index′ represents the substituted carbon; Contours numbered and colored in cyan with mark index′′ represents carbon adjacent to substituted position.

The water soluble cellulose derivative (sample 4, Table 1) was characterized by ^1^H, ^13^C and HSQC NMR spectroscopy spectra (Figs 1, 2, 3) acquired in D_2_O. The possible assignments of the peaks shown in NMR spectra were made according to previously reported data^25^.

The ^1^H NMR spectrum showed that the modification of cellulose with dichloroacetyl chloride in EmimAc yielded acetates at positions C-3, C-2 and C-6 respectively (Fig. 1; peaks numbered and colored in purple and marked with index′). The signals for the protons in substituted C-6 were identified at 4.56 ppm for H6s-a and at 4.23 ppm for H6s-b as a result of the modification. Moreover, the proton signals from the substituted position C-3 (4.99 ppm) and C-2 (4.70 ppm, overlapped by solvent peak) could be detected. It was noted that the signals at 4.62 ppm (C-1 adjacent to substituted C-2), 3.46 ppm (C-2 adjacent to substituted C-3), 3.41 ppm (C-3 adjacent to substituted C-2) and 3.83 ppm (C-4 adjacent to substituted C-3) were also detected (peaks colored in cyan and marked with index′′).

As shown in ^13^C NMR spectrum (Fig. 2), no signals for the imidazole were detected, revealing the residual ionic liquid was completely removed. The three carbonyl carbon signals observed at 173.9, 173.0 and 169.6 ppm were assigned to the acetates at positions C-2, C-3 and C-6, respectively^26^. Moreover, signals around 20.3 ppm for the methyl moiety of the acetyl group were observed, indicating the formation of cellulose acetates. As shown from HSQC NMR spectra (Fig. 3), about half of the hydroxyl group on C6 position in this water-soluble cellulose acetate have not been substituted. According to the semi-quantitative analysis methods by integrating the substituted positions, the relative degree of substitution in C-6, C-3 and C-2 was in the ratio of 14.1: 3.7: 1.0, which could be concluded that the relative reactivity of hydroxyl groups in cellulose was in the order C-6>C-3>C-2, which was inconsistent with previous literature^27^.

**Figure 4:**
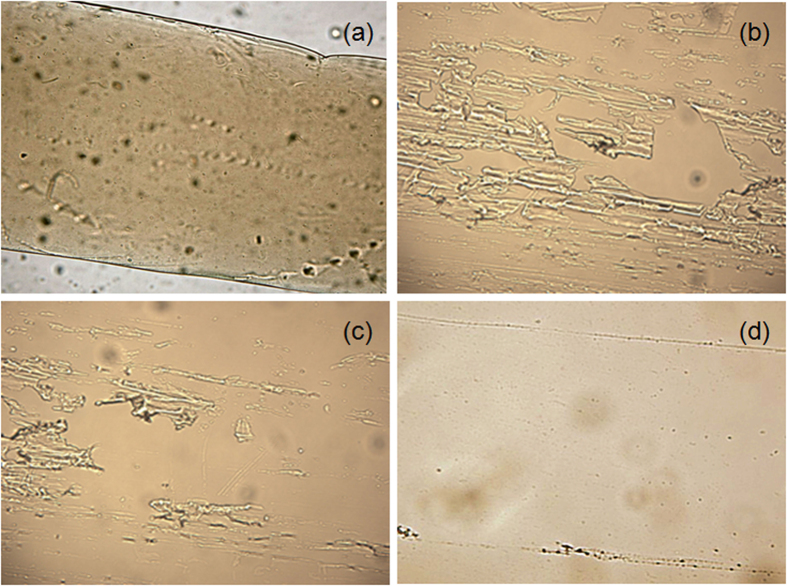
polarizing microscope images of the WSCA dissolution process: (**a**) cellulose derivative sample in ethanol; (**b**) cellulose derivative sample in water after 5 s; (**c**) cellulose derivative sample in water after 30 s; (**d**) cellulose derivative sample in water after 60 s.

The dissolution process of the modified cellulose in water was monitored by polarizing microscope as shown in Fig. 4. As a control, a thin film of modified cellulose sample was soaked in ethanol that does not dissolve the sample (Fig. 4a). When soaked in water, the cellulose acetate film started to breakdown into smaller pieces (Fig. 4b). The dissolution process was very fast, above 50% of the sample was dissolved in 30 s (Fig. 4c) and completely dissolved in 60 s (Fig. 4d).

**Figure 5:**
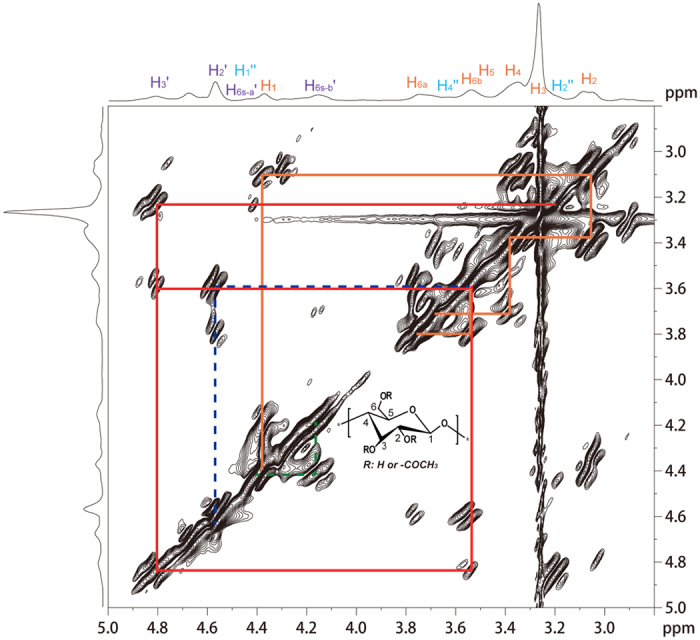
COSY NMR spectra of sample 4 (DS = 0.63) recorded in DMSO-*d*_6_ at 60 °C. Assigned cross-peaks: —cross-peaks colored in rust represents the unsubstituted unit or partially substituted in glucose units; —cross-peaks colored in red represents the unit 3, 6-di-*O*-acetyl cellulose; —cross-peaks colored in blue represents the unit 2, 6-di-*O*-acetyl cellulose; —cross-peaks colored in green represents the unit 6-mono-*O*-acetyl moiety.

It was reported that the water solubility of the CA depends not only on the total DS but also on the distribution of substituents in the AGU^28^. The substitution of C-2 and C-3 secondary hydroxyl groups is indispensable for the water solubility of CA^29^. In order to examine the structural features of the WSCA synthesized in this work, the sample 4 with DS 0.63 was further characterized by ^1^H-^1^H COSY NMR (Fig. 5).

## Discussion

As shown in the COSY NMR spectra, it was evident that the about half of the glucose repeating units were not acetylated (Fig. 5, peak no. 1–6 in rust). All proton-proton correlation signals of this repeating unit could be identified by tracing the cross-peaks; beginning with the H-1 signal at around 4.38 ppm, correlated by the H-2 at about 3.05 ppm and the H-3/H-4 signals at 3.39 ppm. The signal for the protons on C-5 was at 3.41 ppm. The signals for the protons on C-6 were identified at 3.75 ppm for H-6a and 3.55 ppm for H-6b at this position. Notably, considerable amount (about 50%) of hydroxyl groups on C-6 were acetylated (cross-peaks colored in green) and small amount of hydroxyl groups on C-3 or C-2 were acetylated (cross-peaks colored in red or in blue), which was consistent with those results from HSQC characterization (Fig. 3). These results revealed that cellulose was acetylated with the formation of 6-mono-*O*-acetyl moiety, 3,6-di-*O*-acetyl cellulose and 2,6-di-*O*-acetyl cellulose.

The physicochemical properties of obtained WSCA were also characterized by means of FT-IR spectroscopy and XRD. The FTIR spectra of cellulose samples with different reaction time were performed and results are shown in Fig. S1. The introduction of functional groups into the polysaccharide was identified by different bands that can be assigned to C = O stretching in ester group (1732 cm^−1^) and the stretching of (O) C-O (1244 cm^−1^) as known from the literature^20^,^29^. Additionally, the X-ray diffraction (XRD) pattern of original cellulose showed characteristic peaks centered at 2θ = 14.9°, 16.8°, 23.3° and 34.2 for (−110), (110) (200) and (040) reflections planes are characteristic for cellulose I crystal (Fig. S2)^30^. The regenerated control sample showed a broad peak at approximately 2θ at 12.5° and 22.4° with Miller indices of (−110), (110), which is characteristic for the crystalline form of cellulose II^31^. However, the WSCA samples merely presented a wide and plane peak of around 2θ = 20.0 degree, corresponding to (110) crystallographic planes. These results demonstrated the original crystalline structure of cellulose was seriously destroyed during the derivatization process and the crystal structure of the CA samples after modification was the combination of amorphous and the crystalline regions. Interestingly, a broadened peak centered at 5.1 degree was observed from the cellulose sample with reaction times longer than 12 h, which was corresponded to the lateral spaces between the long molecular chains of cellulose produced by functionalization of the glucopyranose rings (002) plane^32^,^33^, revealing successful esterification of cellulose achieved in EmimAc with Cl_2_AcCl as impeller. The appeared lateral spaces could readily absorbed water molecular and facilitating the dissolution of cellulose in water.

**Figure 6:**
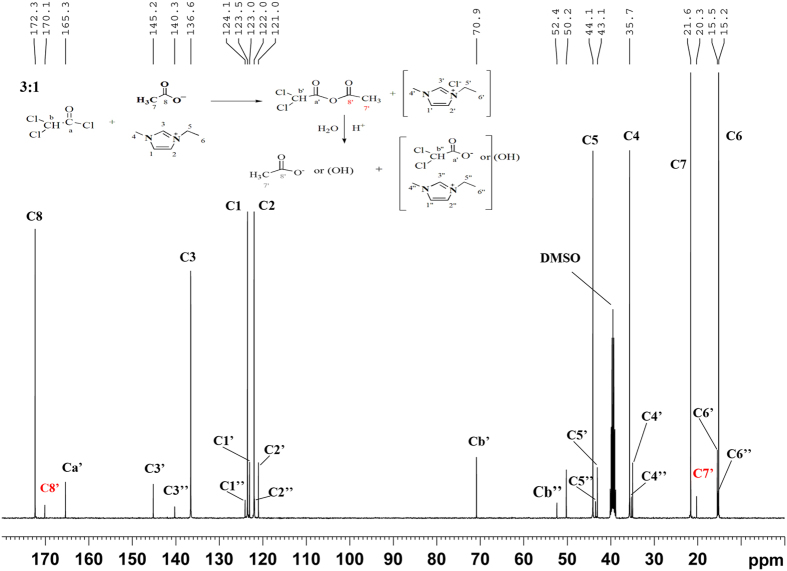
^13^C NMR spectra of the mixture of EmimAc/Cl_2_AcCl measured in DMSO-*d*_6_. (Molar radio 3:1).

As discussed above, modification of cellulose with dichloroacetyl chloride (Cl_2_AcCl) in EmimAc produced, surprisingly, the water-soluble cellulose acetates. In order to evaluate the role of EmimAc, one cellulose derivative was made in dimethylacetamide/lithium chloride (DMAc/LiCl) with Cl_2_AcCl. From the element analysis, it is apparently that this product was cellulose dichloroacetate with DS value of 0.07. The extremely low DS of this cellulose derivative was obviously the result of not using any base like pyridine. Therefore, the ionic liquid EmimAc used here was not just as cellulose solvent but also a “promoter” that helps make higher DS cellulose acetate products. Elemental analysis suggested that the cellulose derivative products made in EmimAc contained only trace amount of chlorine whereas significant amount (4.5%) of chlorine was found in sample 7 made in DMAc/LiCl solvent. It was reported that the ionic liquid solvent EmimAc can act as acetylating agent when other acylating agents such as 2-furoyl chloride, *p*-toluenesulfonyl chloride or trityl chloride are present, producing cellulose acetates^34^. To understand whether the Cl_2_AcCl plays the same role as those acylating agents such as 2-furoyl chloride, *p*-toluenesulfonyl chloride reported before where EmimAc was used as cellulose solvent for chemical modification of cellulose, the mixture of EmimAc/Cl_2_AcCl (molar ratio 3:1) without cellulose was analyzed by ^13^C NMR spectra in DMSO-*d*_6_ (Fig. 6), and the ^1^H NMR and HSQC spectra of the mixture are presented in Figs S3 and S4.

**Figure 7:**
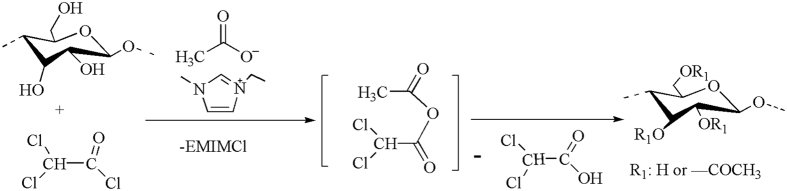
General synthetic route to WSCA.

As seen from Fig. 6, the new signal at 170.1 ppm and 20.3 ppm (marked in red), which were attributed to carbonyl and methyl moiety of the mixed anhydride. These result indicated the formation of mixed dichloroacetyl acid/acetic acid anhydride. Meanwhile, the EmimAc was partially converted into EmimCl as shown in this spectrum. Based on this observation, it could be concluded that the Cl_2_AcCl reacts with EmimAc preferentially forming the mixed anhydride that then reacts with the dissolved cellulose producing cellulose acetate product. Consequently, the final products obtained was cellulose acetate instead of the dichloroacetate derivatives expected initially. In addition, more weak peaks detected in Fig. 6 was presumably induced by the hydrolysis of the mixed anhydride. The general synthetic route was summarized in Fig. 7. Moreover, in order to further confirm the mechanism of the reaction, two more acylating reagents, chloroacetyl chloride (ClAcCl) and benzoyl chloride, were mixed with EmimAc respectively to test if the mixed anhydride intermediates can also be produced. As seen in Figs S5 and S6, new peaks attributed to mixed anhydride were all detected, revealing the formation of mixed anhydride in both cases. Although the reaction mechanism is the same, the products obtained by chloroacetyl and benzoyl chloride couldn’t solubilize in water. This comparison indicated that the mixed dichloroacetyl acid/acetic acid anhydride is irreplaceable for preparation of water-soluble CA.

### Dissolution mechanism

**Figure 8:**
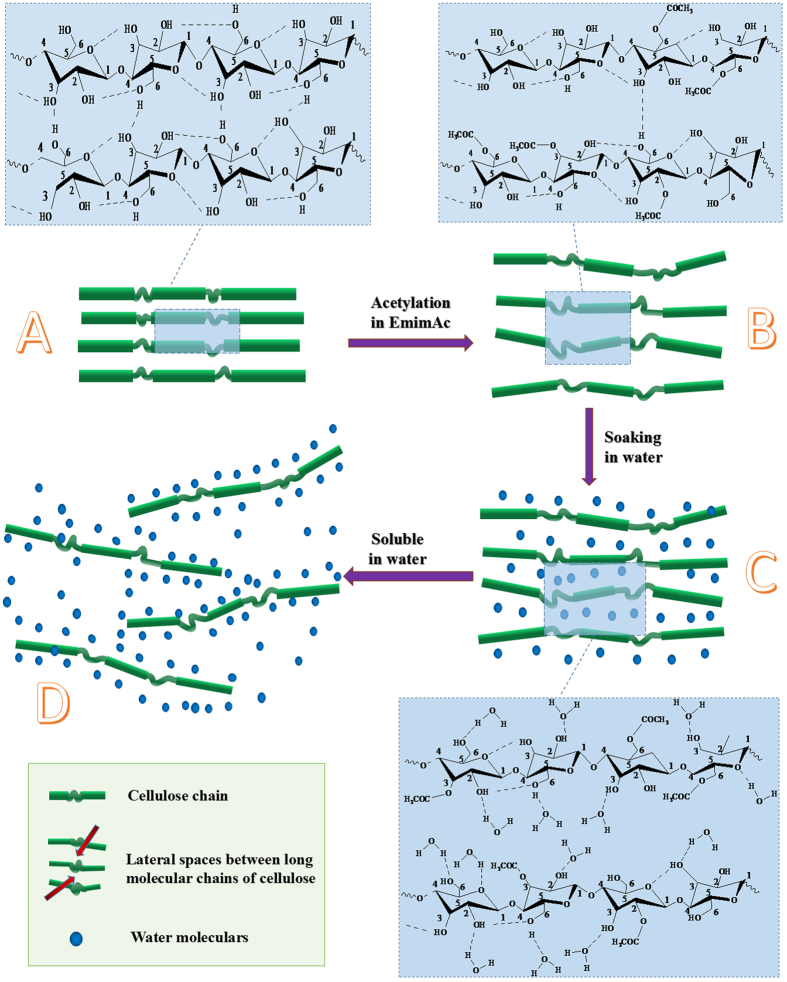
(**A**) molecular structure and hydrogen bonding network of natural cellulose; (**B**) structure of cellulose after acetylation in EmimAc, the hydrogen bonding network was partially destroyed, the crystal structure was the combination of amorphous and the crystalline regions and lateral spaces between long molecular chains of cellulose was appeared; (**C**) hydrogen bonds between water molecular and hydroxyl groups of cellulose were formed and the hydrogen bonding network was completely destroyed; (**D**) CA sample was soluble in water.

It is well known that the insolubility of cellulose is mainly attributed to the strong inter and intramolecular hydrogen bonding network and its crystalline structure. Herein, the destruction of hydrogen bonding networks and change its crystalline structure is the key point for improving the solubility of cellulose. As shown in Fig. 8A, complicated hydrogen bonding network existed in natural cellulose, the C-3 and C-6 hydroxyl groups play a key role in intramolecular hydrogen bonds, especially hydroxyl groups at C-3^35^. Gagnaire *et al*.^36^ has claimed that the intramolecular hydrogen bonding between the hydroxyl group at C-3 position and a heterocyclic oxygen atom in neighboring glucopyranose unite of cellulose may exist even in the solution. Thus, in order to make the cellulose soluble in water or aqueous solutions, the hydroxyl group at C-3 position must be partially substituted to destroy the strong inter and intramolecular hydrogen bonds. For the water-soluble CA sample in this study, base on the semi-quantitative analysis methods by integrating the substituted positions in C-6, C-3 and C-2 (HSQC NMR spectra Fig. 3), a significant amount of hydroxyl group at C-3 position were accurately substituted, which is the crucial factor for the water-solubility of WSCA samples. On the other hands, the introduce of a acetyl substitution at C-6 position destroyed the intermolecular hydrogen bonds between the hydroxyl group at C-6 position and a heterocyclic oxygen atom in neighboring glucopyranose unite, resulting in destruction of the intermolecular hydrogen bonds (Fig. 8B). Furthermore, the substitution at C-6 position could also widen the distance between long molecular chains of cellulose^37^ (Fig. 8B), this lateral spaces were accurately detected by XRD spectra (Fig. S2). When the CA samples were immersed in water, the wide space between molecular chains could readily absorbed water molecular, and the small water molecular could easily reach the surface of the cellulose molecule chain. Owing to the extremely low DS, enormous free hydroxyl groups were exposed to water molecules, hydrogen bonds between hydroxyl groups and water molecules formed quickly, leading to the break of the hydrogen bonding networks (as shown in Fig. 8C). As the swelling reaction proceeds, the hydrogen bonding network was completely destroyed and the cellulose molecular chains was stripped by water molecules, resulting in the soluble of cellulose in water (Fig. 8D). It’s interesting to note that the acetyl group of CA is less hydrophilic than hydroxyl group, herein, the affinity of the substituent group against water seemed another important factor.

In summary, the modification of cellulose using dichloroacetyl chloride (Cl_2_AcCl) in 1-ethyl-3-methylimidazolium acetate (EmimAc) produced cellulose acetate unexpectedly. It was demonstrated by NMR spectroscopy that the acetylation of cellulose was achieved by the mixed anhydride formed from a reaction between dichloroacetyl chloride (Cl_2_AcCl) and acetate group of EmimAc. Amazingly, the obtained cellulose acetate with relatively higher DP values in the range from 650 to 680 possessed good water solubility. Based on the 2D NMR characterization, it was found that 6-mono-*O*-acetyl moiety, 3,6-di-*O*-acetyl cellulose and 2,6-di-*O*-acetyl cellulose were all synthesized during this homogeneous functionalization. However, it should be cautioned that the consumption of the acetate anion of EmimAc could be a problem when considering the recycling and reusing of this ionic liquid.

## Methods

### Material and chemicals

Cotton linter (degree of polymerization, DP = 850) was kindly supplied by Silver Hawk Fiber Corporation (Shandong province, China). Ionic liquid, 1-ethyl-3-methylimidazolium acetate (EmimAc, ≥95%), was purchased from Shanghai Cheng Jie Chemical Co., Ltd. Dichloroacetyl chloride (Cl_2_AcCl, 97%) was obtained from Alfa Aesar (A Johnson Matthey Company). Chloroacetyl chloride (ClAcCl, ≥99.0%) was purchased from Sinopharm Chemical Reagent Co., Ltd. and benzoyl chloride (≥98.0%) was purchased from Guangzhou Guanghua Chemical Factory Co., Ltd. All chemicals were analytical grade reagents and used as received without further purification.

### Reaction of cellulose with Cl_2_AcCl in EmimAc

1.00 g cellulose (6.17 mmol with respect to the repeating unit) was dissolved in 30 g EmimAc under magnetic stirring for 30 min at 80 °C until the cellulose was dissolved completely to yield a 3 wt% solution. Then, the solution was cooled to 70 °C, Cl_2_AcCl 3.91 g (the molar ratio of reactants, i.e., dichloroacetyl chloride/anhydroglucose unit (AGU) was 3:1) was added to the cellulose solution dropwise under stirring. The reaction mixture was stirred for 24 h. Subsequently, the hot solution was poured in 200 mL ethanol, and then the modified cellulose acetate was regenerated and precipitated in the ethanol bath. After that, the modified cellulose acetate was separated by filtration and soaked in 200 mL methanol under stirring for 24 h to remove any residual ionic liquid and unreacted reagents. The product were dissolved in water and dried by freeze-drying.

Yield: 95%, DS = 0.63, determined by ^1^H NMR spectroscopy.

Elemental analysis: C 40.67%, H 6.53%, O 41.13%, Cl 0.034%.

FT-IR (cm^−1^): 3416 *ν*(OH), 1732 *ν*(C = O ester), 1244 *ν*((O) C-O, ester), 1023 *ν*(C-O-C AGU).

^1^H NMR (400 MHz, D_2_O) δ/ppm: 3.2-5.0 (AGU-protons), 3.3 (H-2), 3.5-3.8 (H-3, H-4, H-6b and H-5), 3.9 (H-6a), 4.5 (H-1), 1.8-2.1 (OCOCH_3_).

^13^C NMR (400 MHz, D_2_O) δ/ppm: 173.9 (C = O), 102.6 (C-1), 78.6 (C-4), 74.9 (C-3), 74.0 (C-2), 72.9 (C-5), 62.7 (C-6 substituted), 59.9 (C-6 non-substituted), 20.3 (OCOCH_3_).

### Characterizations

The DP of all the cellulose samples were measured by TAPPI test method as previous report^19^. The crystallinity of the sample was determined by X-ray diffraction (XRD) patterns (XRD-6000, Shimidzu, Japan). All samples were recorded from 5^o^ to 40^o^ (2θ) with a scanning speed of 5^o^ min^−1^. Elemental analysis of samples were conducted by the Ion Chromatography (ICS-2100, Thermo, USA). The Fourier transform infrared (FTIR) spectra of all the samples were measured on a Tensor 27 infrared spectrum instrument (Bruker, German) over the frequency range of 4000 to 400 cm^−1^ at 2 cm^−1^ resolution and 32 scans per sample. Structure characterization was analyzed by NMR spectroscopy on a Bruker AV III 400 MHz and 600 MHz spectrometer and the concentration of sample was 20 mg mL^−1^ in D_2_O. The dissolution process of cellulose derivatives was observed by a polarizing microscope (BX51, OLYMPUS, Japan).

## Additional Information

**How to cite this article**: Pang, J. *et al*. Synthesis of Highly Polymerized Water-soluble Cellulose Acetate by the Side Reaction in Carboxylate Ionic Liquid 1-ethyl-3-methylimidazolium Acetate. *Sci. Rep.* **6**, 33725; doi: 10.1038/srep33725 (2016).

## Supplementary Material

Supplementary Information

## Figures and Tables

**Table 1:** Conditions for and analysis of the modified cellulose.

NO.	Reaction conditions			Products				
	Solvent	Time (h)	Temp. (°C)	DS	Solubility		Cl %	DP
					DMSO	H_2_O		
1	EmimAc	4	70	−	−	−	0.0361	680
2	EmimAc	6	70	0.30	+	+	0.0423	670
3	EmimAc	12	70	0.56	+	+	0.0335	665
4	EmimAc	24	70	0.63	+	+	0.0451	650
5	EmimAc	12	45	0.30	+	+	0.0310	670
6	EmimAc	24	45	0.50	+	+	0.0343	660
7	DMAc/LiCl	6	70	0.07	−	−	4.5030	−

DS: Degree of substitution calculated from ^1^H NMR data. DMSO: dimethylsulfoxide, H_2_O: deionized water, +: soluble, −: insoluble.
