# Supplementary material for: Synthesis of Highly Polymerized Water-soluble Cellulose Acetate by the Side Reaction in Carboxylate Ionic Liquid 1-ethyl-3-methylimidazolium Acetate
Source: Sci Rep. 2016 Sep 20;6:33725. doi: 10.1038/srep33725 (PMC5028776; doi:10.1038/srep33725)
Supplement: Supplementary Information [file srep33725-s1.doc]

**Synthesis of Highly Polymerized Water-soluble Cellulose Acetate by the Side Reaction in Carboxylate Ionic Liquid 1-ethyl-3-methylimidazolium Acetate**

Jinhui Pang, Xin Liu, Jun Yang, Fachuang Lu, Bo Wang, Feng Xu, Mingguo Ma, Xueming Zhang


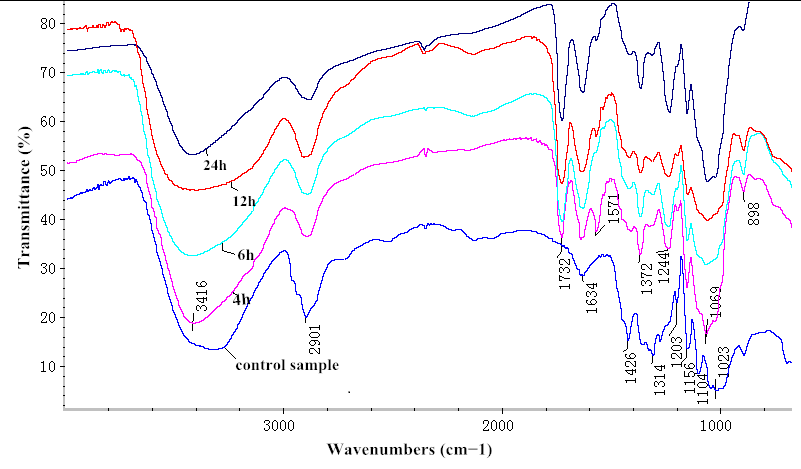


**Fig. S1 FT-IR spectra of unmodified cellulose (control sample) and the WSCA samples with different reaction time (4h, 6h, 12h, 24h) at 70** º**C**


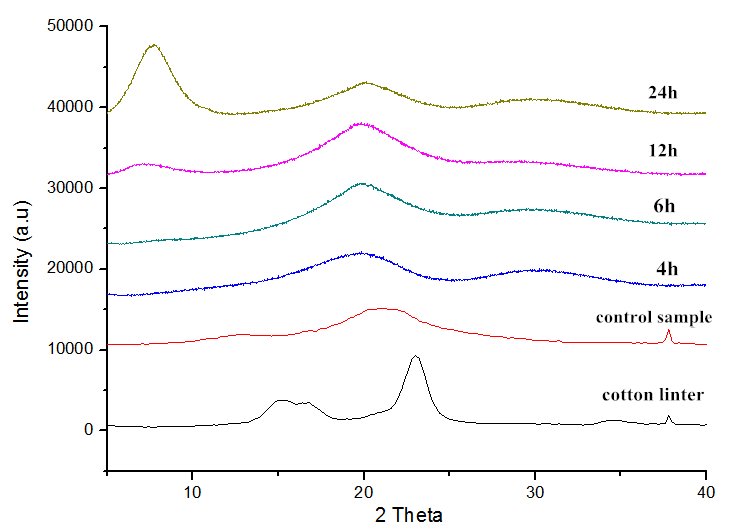


**Fig. S2 X-ray diffraction of cotton linters, unmodified cellulose (control sample), and the WSCA samples with different reaction times (4h, 6h, 12h, 24h).**

**
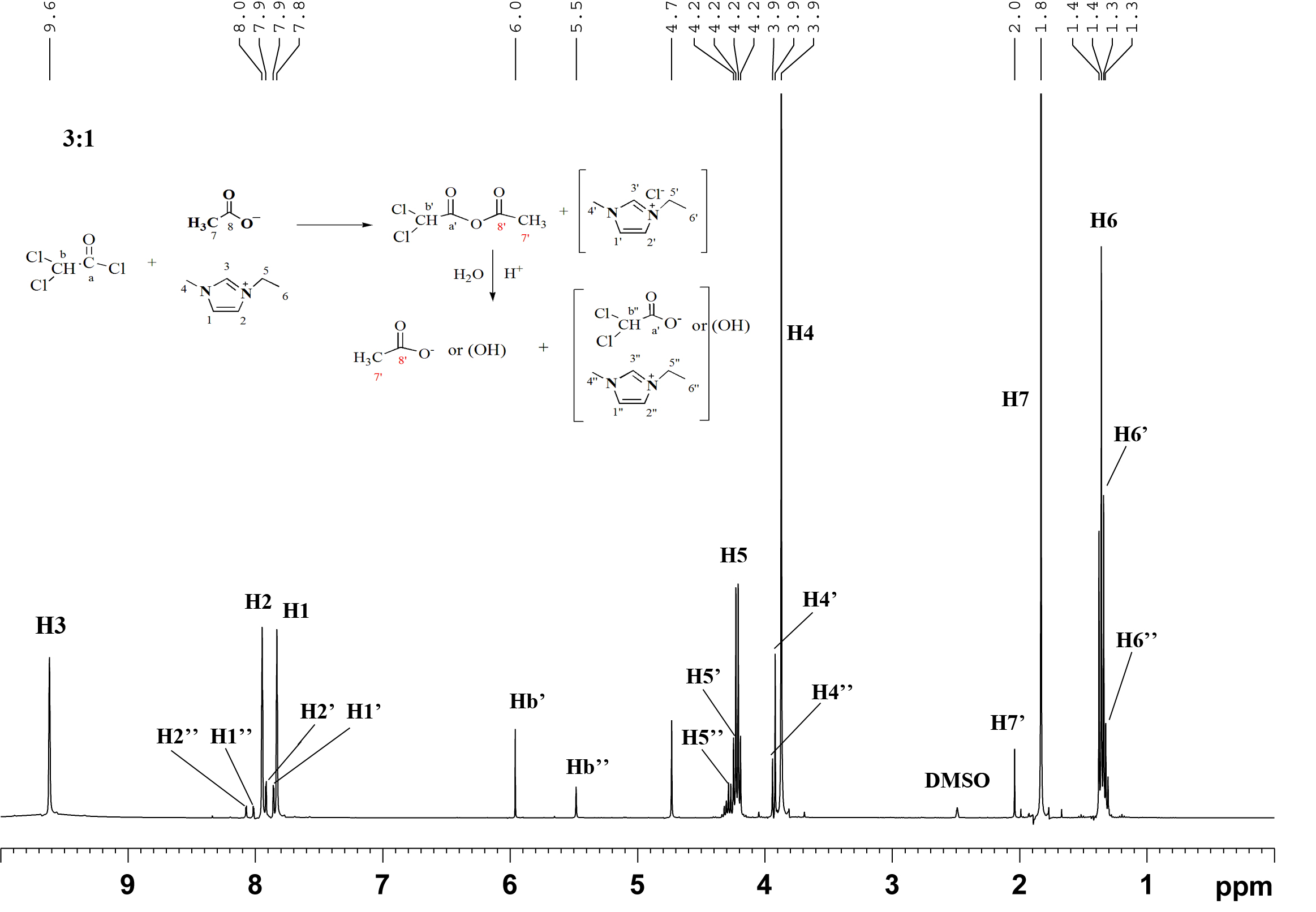
**

**Fig. S3 ^1^H NMR spectra of the mixture of EmimAc/Cl_2_AcCl measured in DMSO-*d*_6_. (Molar ratio 3:1)**

**
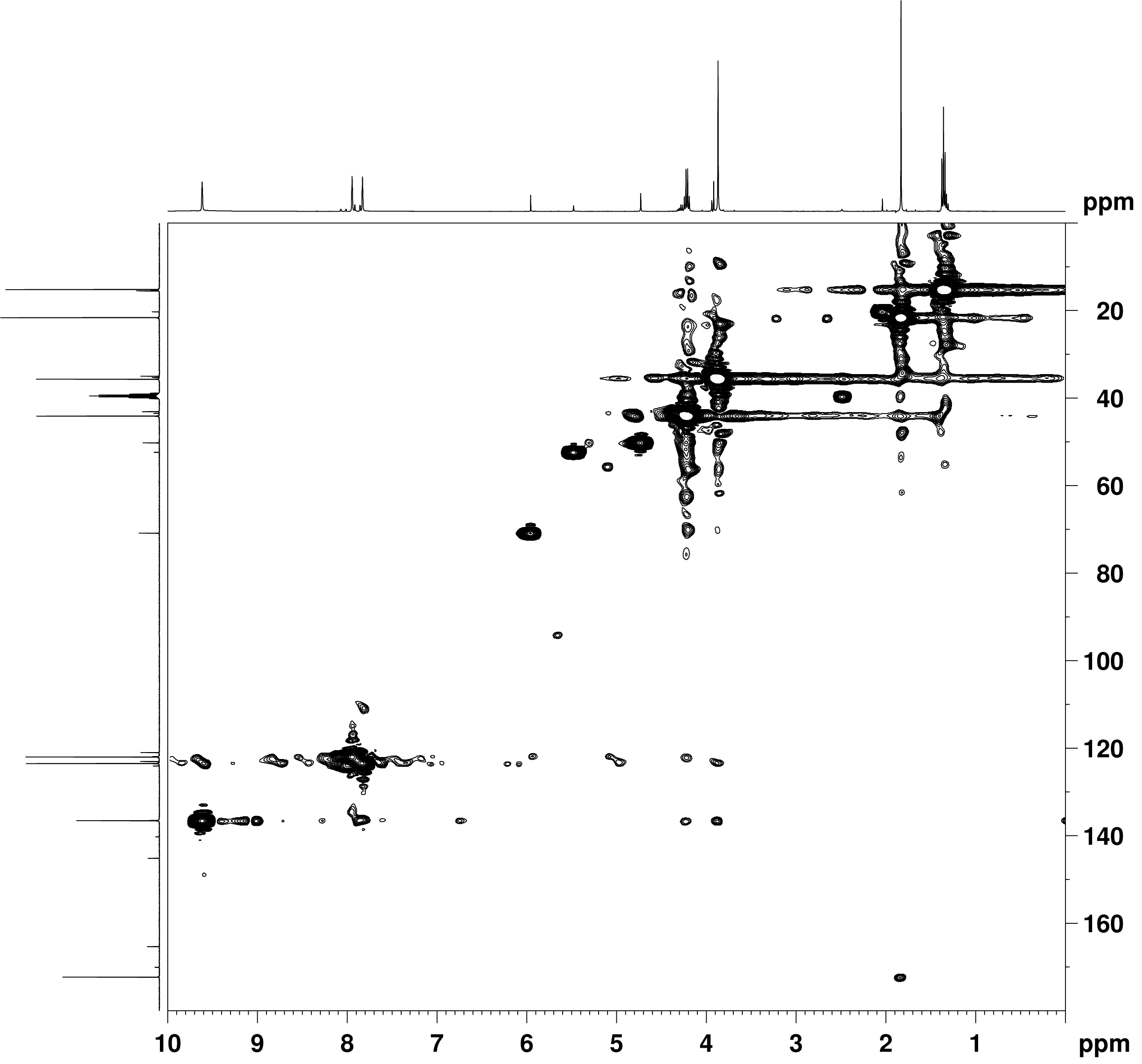
**

**Fig. S4 HSQC spectra of the mixture of EmimAc/Cl_2_AcCl measured in DMSO-*d*_6_. (****Molar ratio 3:1)**

**Fig. S5 ^13^C NMR spectra of the mixture of chloroacetyl chloride/** **EmimAc measured in DMSO-*d*_6_ at 25 ºC.**


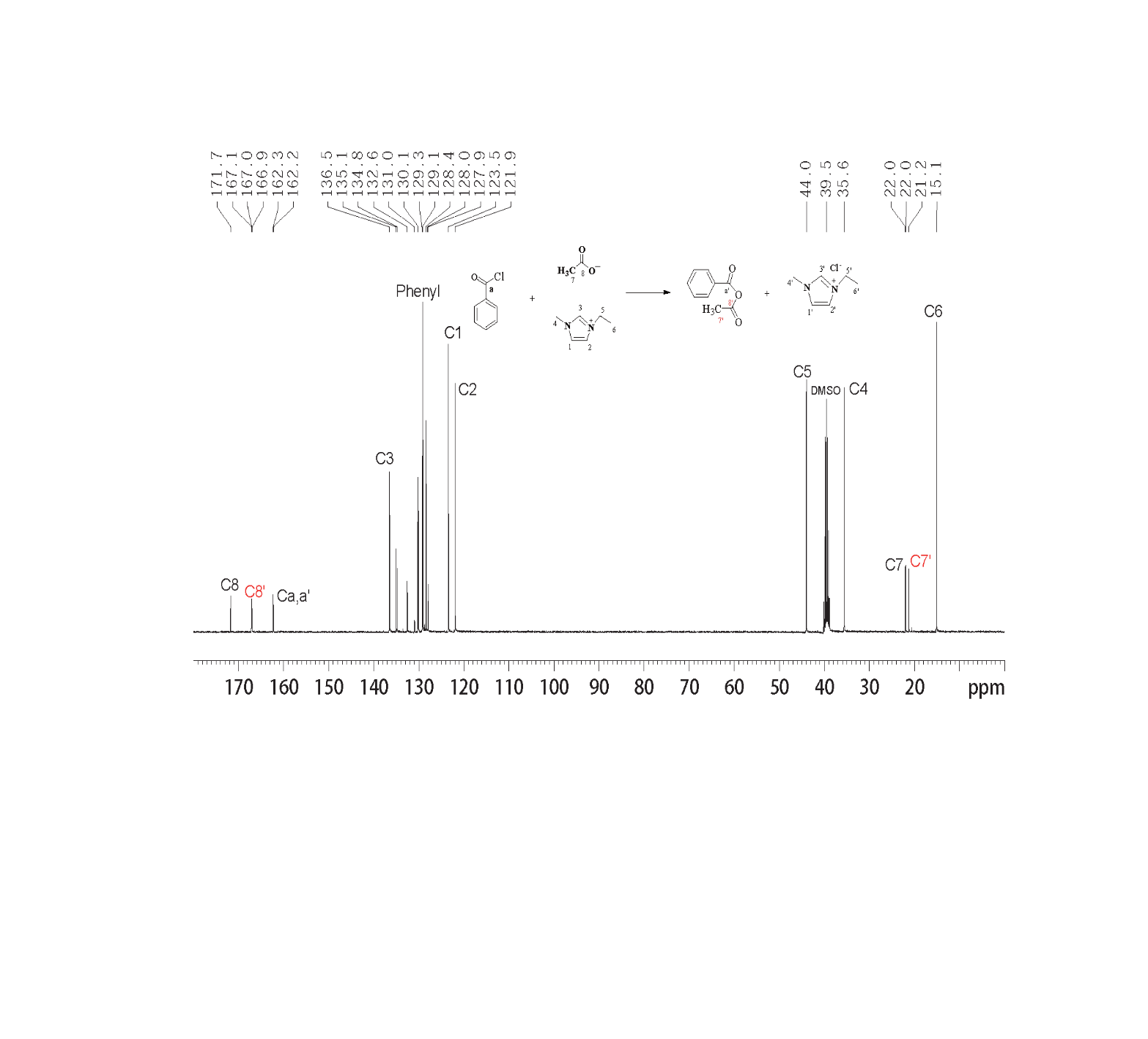


**Fig. S6 ^13^C NMR spectra of the mixture of** **Benzoyl chloride/** **EmimAc measured in DMSO-*d*_6_ at 25 ºC.**
